# Classification of eastward propagating waves on the spherical Earth

**DOI:** 10.1002/qj.3025

**Published:** 2017-04-24

**Authors:** Chaim I. Garfinkel, Itzhak Fouxon, Ofer Shamir, Nathan Paldor

**Affiliations:** ^1^ Fredy and Nadine Herrmann Institute of Earth Sciences The Hebrew University of Jerusalem Israel

**Keywords:** equatorial Kelvin wave, equatorial inertia–gravity waves, shallow‐water waves, Kelvin waves on a sphere

## Abstract

Observational evidence for an equatorial non‐dispersive mode propagating at the speed of gravity waves is strong, and while the structure and dispersion relation of such a mode can be accurately described by a wave theory on the equatorial β‐plane, prior theories on the sphere were unable to find such a mode except for particular asymptotic limits of gravity wave phase speeds and/or certain zonal wave numbers. Here, an ad hoc solution of the linearized rotating shallow‐water equations (LRSWE) on a sphere is developed, which propagates eastward with phase speed that nearly equals the speed of gravity waves at all zonal wave numbers. The physical interpretation of this mode in the context of other modes that solve the LRSWE is clarified through numerical calculations and through eigenvalue analysis of a Schrödinger eigenvalue equation that approximates the LRSWE. By comparing the meridional amplitude structure and phase speed of the ad hoc mode with those of the lowest gravity mode on a non‐rotating sphere we show that at large zonal wave number the former is a rotation‐modified counterpart of the latter. We also find that the dispersion relation of the ad hoc mode is identical to the n = 0 eastward propagating inertia–gravity (EIG0) wave on a rotating sphere which is also nearly non‐dispersive, so this solution could be classified as both a Kelvin wave and as the EIG0 wave. This is in contrast to Cartesian coordinates where Kelvin waves are a distinct wave solution that supplements the EIG0 mode. Furthermore, the eigenvalue equation for the meridional velocity on the β‐plane can be formally derived as an asymptotic limit (for small (Lamb Number)^‐1/4^) of the corresponding second order equation on a sphere, but this expansion is invalid when the phase speed equals that of gravity waves i.e. for Kelvin waves. Various expressions found in the literature for both Kelvin waves and inertia–gravity waves and which are valid only in certain asymptotic limits (e.g. slow and fast rotation) are compared with the expressions found here for the two wave types.

## 1 Introduction

On the equatorial *β*‐plane the elegant theory developed by Matsuno (1966, M66 hereafter), which is repeated in Holton and Lindzen (1968), has two types of eastward propagating waves: inertia–gravity (also known as Poincaré) waves with countably infinite modes: *n* = 0, 1, 2… (denoted hereafter as EIGn), and the non‐dispersive and single mode (i.e. they have only one meridional mode number, *n*) Kelvin waves. The properties of Kelvin waves that distinguish them from EIGn waves are: (i) they are non‐dispersive with phase speed that equals the phase speed of gravity waves at all zonal wave numbers; (ii) the meridional velocity component vanishes identically so the zonal component and the free surface alone solve the three scalar linear rotating shallow‐water equations (LRSWE hereafter); and (iii) they are derived as a degenerate (special) solution of the LRSWE instead of solutions of an associated eigenvalue equation, i.e. these waves belong to the null‐space of the associated eigenvalue equation. These properties also typify Kelvin waves in midlatitudes (Cushman‐Roisin, 1994; Vallis, 2006; Paldor *et al*., 2007).

In spherical coordinates the LRSWE are much more complex than in Cartesian coordinates since in addition to the nonlinear latitude dependence of the Coriolis frequency, *f* = 2*Ω*sin*ϕ* (where *Ω* is the frequency of Earth's rotation about its axis and *ϕ* is the latitude), the vectorial operators div (in the continuity equation) and grad (in the momentum equations) are also latitude‐dependent. The complexity of LRSWE in spherical coordinates can be appreciated by comparing the concise and clear theory of zonally propagating waves on the unbounded equatorial *β*‐plane in M66 with its counterpart on a sphere developed in the seminal work of Longuet‐Higgins (1968, L‐H hereafter). While in the planar theory the problem straightforwardly reduces to the well‐known Schrödinger eigenvalue problem of a harmonic oscillator of quantum mechanics with no free parameters, on a sphere the eigenvalue problem is too cumbersome, and approximate analytic expressions can be obtained only in asymptotic limits, e.g. when sin*ϕ* ≈ *ϕ* ≪ 1 or for slow/fast rotation. Previous attempts at developing an analytic solution on the sphere that mimics the planar Kelvin wave solution derived by Matsuno raise several questions. In L‐H theory the free non‐dimensional parameter is Lamb number *ϵ* = (2*Ωa*)^2^/*gH* where *a* is the planet's radius, *g* is the gravitational acceleration and *H* is the thickness of the layer of fluid so (*gH*)^½^ is the gravity wave phase speed. In the limit of small *ϵ* (i.e. slow rotation or thick layer) L‐H finds (see Chapter 4) only one class of eastward propagating waves (denoted as class 1) while in the large *ϵ* limit (i.e. fast rotation or thin layer) there are two types of such waves (denoted as type 1 and type 3). The type‐3 wave of the large *ϵ* limit is completely missing from the small *ϵ* limit which raises the issue of how can the non‐dispersive type‐3 wave vanish when *ϵ* (i.e. the planet's rotation frequency, *Ω*) is decreased? Boyd and Zhou (2008, BZ hereafter) developed an asymptotic, large *ϵ*, theory for an ad hoc mode that is similar to L‐H's type‐3 mode but is not limited to O(1) zonal wave number as in L‐H theory. The meridional variation of BZ's ad hoc mode is Gaussian as in L‐H's type‐3 wave but the relationship between this mode and the other waves/modes found in L‐H is unclear.

Given the strong observational evidence (gained from several independent data sources using different methods) for a wave that closely resembles Matsuno's planar wave solution (Wallace and Kousky, 1968; Wheeler and Kiladis, 1999; Randel and Wu, 2005; Kiladis *et al*., 2009), it is reasonable to suspect that the LRSWE can support such a mode on a sphere for the parameter range relevant to Earth, and this work confirms that this is indeed the case.

Recently, Schrödinger eigenvalue problems were formulated for zonally propagating wave solutions of the LRSWE in midlatitudes (Paldor *et al*., 2007; Paldor and Sigalov, 2008) and approximate Schrödinger eigenvalue problems were also formulated on a sphere (De‐Leon and Paldor, 2011; Paldor *et al*., 2013; Paldor, 2015). These formulations provide a clear and concise way of identifying the various waves and modes that solve the LRSWE. In the present study we derive an ad hoc wave solution of the LRSWE on a sphere that resembles both L‐H's type‐3 wave (in the fast rotation limit) and BZ's ‘Kelvin’ wave. We classify this ad hoc wave solution by analysing the solutions of a corresponding Schrödinger eigenvalue problem. In particular, we examine how L‐H's type‐3 wave and BZ's ‘Kelvin’ wave differ from M66's Kelvin wave and the relation between these spherical ‘Kelvin’ waves and the EIGn modes.

This work is organized as follows: in sections 2 and 3 we present the dilemma that faces any attempt to derive a Kelvin‐like solution on the sphere and show that the standard, text‐book techniques that succeed on the plane fail when applied to the sphere. In the rest of the article we demonstrate that on a sphere there are no special solutions that supplement the EIGn modes and that the EIG0 mode shares some of the properties of the planar Kelvin wave. In section 4 we develop an ad hoc eastward propagating nearly non‐dispersive wave solution without specifying the type of this wave and compare this mode to M66's Kelvin wave. In section 5 we derive and solve an eigenvalue Schrödinger equation whose numerable energy levels yield a clear classification of the various modes of the LRSWE. In section 6 we describe the numerical method for finding numerical solutions of the exact LRSWE and these solutions are interpreted in section 7 in terms of the ad hoc solution of section 4 and the analytic solutions of the eigenvalue Schrödinger equation derived in section 5. We discuss our results in the context of previous solutions of the shallow‐water equations on a sphere in section 8 and conclude the article in section 9.

## 2 A new perspective on the degeneracy of Kelvin waves on a rotating plane

In order to more fully motivate our study, we begin by posing the question: why does the derivation that produces a unique Kelvin wave mode on the plane fail when applied to the sphere? To answer this question, we review the derivation of LRSWE modes on the plane and consider the differences when a comparable derivation is attempted on the sphere. The derivation of Kelvin waves on the *f*‐plane in all textbooks on the subject (e.g. Pedlosky, 1987; Cushman‐Roisin, 1994; Vallis, 2006) begins by setting the meridional velocity component equal to zero and the same is true for the equatorial *β*‐plane (M66). This approach will be followed in the next section (i.e. section 3) but a more general perspective can be developed that highlights the difference between Cartesian and spherical coordinates.

In Cartesian coordinates where (*x*, *y*) and (*u*, *v*) are the coordinates and velocity components in the (east, north) directions, respectively, the LRSWE are:

(1) $$\begin{array}{c} \frac{\partial u}{\partial t}-fv=-g\frac{\partial \eta }{\partial x},\\ \frac{\partial v}{\partial t}+fu=-g\frac{\partial \eta }{\partial y},\\ \frac{\partial \eta }{\partial t}=-H\left(\frac{\partial u}{\partial x}+\frac{\partial v}{\partial y}\right)\text{,} \end{array}$$

where $f=f_{0}+\beta y=2\Omega \sin\phi_{0}+\frac{2\Omega \cos\phi_{0}}{a}y$ is the Coriolis parameter, *η* is the deviation of the free surface height from its mean value *H* (so the thickness of the layer of fluid is *h* = *H* + *η*) and *t* is time. To simplify the analysis that follows we scale the velocity components on 2*Ωa*, time on (2*Ω*)^−1^, *η* on *H* and *x* and *y* on *a*. Assuming that *u*, *v* and *η* are described by a zonally propagating wave e^i(*kx−ωt*)^ (in which *ω* is the wave's frequency and *k* is its zonal wave number so *C* = *ω*/*k* is the phase speed) with *y*‐dependent amplitudes, we use the first equation to express *u* as a linear combination of *V* and *η* so it can be eliminated from the other two equations. Defining *V*(*y*) = i*v*(*y*)/*k* then yields the second‐order (in *y*) system:
(2)
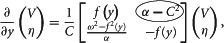



where the non‐dimensional Coriolis parameter is: *f*(*y*) = sin *ϕ*_0_ + *y* cos *ϕ*_0_ and *α* = *gH*(2*Ωa*)^−2^ is the inverse of the Lamb number (denoted as *ϵ* in L‐H). Since the only assumption made in transforming (1) to (2) is that the solutions vary in *x* and *t* as e^i*k*(*x−Ct*)^, Kelvin waves should satisfy Eq. (2). Indeed, for *C* ^2^ = *α* the term on the second column of the first row (marked by a circle) vanishes identically so *V* decouples from *η* and satisfies a first‐order equation only. Thus, in order for *V* to vanish at two boundaries (e.g. two channel walls or *y* = ±∞) it has to vanish identically. With *V*(*y*) ≡ 0, *η* has to satisfy the homogenous first‐order equation

(3) $$\frac{\partial \eta }{\partial y}=-\frac{\sin\phi_{0}+y\cos\phi_{0}}{C}\eta$$

with no accompanying boundary conditions. This degeneracy of Kelvin waves is independent of the form assumed for *f*(*y*) so the solutions exist on the midlatitude *f*‐/*β*‐plane as well as on the equatorial *β*‐plane, i.e. the equatorial theory of M66 is a particular case of this general outline when *f*(*y*) = *βy*.

The other two wave types, inertia–gravity (Poincaré) waves and planetary (Rossby) waves, are derived from the second‐order eigenvalue equation obtained from system (2) by differentiating the *V*‐equation with respect to *y* and eliminating both *η* and ∂*η*/∂*y* from the resulting equation (Paldor *et al*., 2007).

We now follow the same steps in spherical coordinates, and demonstrate that such degeneracy does not exist on a sphere. In spherical coordinates where *λ* and *ϕ* are the longitude and latitude, respectively, *u* and *v* are the zonal and meridional velocity components, respectively, and *η*, *H* and *g* are as in (1), the scalar LRSWE on a sphere of radius *a* are:

(4) $$\begin{array}{c} \frac{\partial u}{\partial t}-fv=-\frac{g}{a\cos\phi }\frac{\partial \eta }{\partial \lambda },\\ \frac{\partial v}{\partial t}+fu=-\frac{g}{a}\frac{\partial \eta }{\partial \phi },\\ \frac{\partial \eta }{\partial t}=-\frac{H}{a\cos\phi }\left(\frac{\partial u}{\partial \lambda }+\frac{\partial \left(v\cos\phi \right)}{\partial \phi }\right)\text{.} \end{array}$$

Scaling the variables as before and assuming that *u*, *v* and *η* all vary in *λ* and *t* as a zonally propagating wave e^i*k*(*λ*−*Ct*)^ (where *k* is the zonal wave number which is a dimensionless integer and *C* is the phase speed in units of radians over time so *ω* = *kC* is the wave's frequency), the zonal momentum equation, i.e. the first equation of system (4), can be employed to express the amplitude of *u* as a linear combination of the amplitudes of *v* and *η*:

(5) $$u=\frac{1}{C\cos\phi }\left(\sin\phi V\cos\phi +\alpha \eta \right)\text{,}$$

where *V* = i*v*/*k*. Substituting this expression for *u* in the other two equations of (4) yields the following second‐order differential set:

(6) $$\begin{array}{cc} & \frac{\partial }{\partial \phi }\left[\begin{array}{c} V\cos\phi \\ \eta \end{array}\right]=\frac{1}{C\cos\phi }\left[\begin{array}{cc} \sin\phi & \left(\alpha -C^{2}\cos^{2}\phi \right)\\ \left(\frac{\omega^{2}-\sin^{2}\phi }{\alpha }\right) & -\sin\phi \end{array}\right]\\ & \;\times \left[\begin{array}{c} V\cos\phi \\ \eta \end{array}\right]\text{.} \end{array}$$

The boundary conditions that *V*cos*ϕ* and *η* have to satisfy are regularity at the (singular) poles, *ϕ* = ±π/2 where cos *ϕ* vanishes. As will be discussed in section 5 and as was shown in De‐Leon and Paldor (2011), Paldor *et al*. (2013) and Paldor (2015), it is possible to form an eigenvalue equation associated with this system whose solutions form a complete set and whose eigenvalues are closely related to the number of zero‐crossings of the eigenfunction. However, the relevant point is immediately apparent by comparing (2) and (6): unlike the circled term in (2) there is no value of *C* for which the corresponding term in the second column on the first line of (6) vanishes identically. Thus, the equations for *V* and *η* in (6) do not de‐couple, and one cannot *a priori* expect to find solutions of (6) other than those that solve the corresponding Schrödinger eigenvalue equation to be developed in section 5.

## 3 The ‘fingerprints’ of Kelvin waves on a sphere

A second possible method (complementary to that shown in section 2) to derive Kelvin waves on a plane is to set *v*(*y*) = 0 identically, and thus we explore how the LRSWE on the sphere behave when we posit this condition, as some insight can be gained by identifying the step at which the derivation fails. Setting *v* = 0 in system (4) yields:

(7) $$\begin{array}{c} \frac{\partial u}{\partial t}=-\frac{g}{a\cos\phi }\frac{\partial \eta }{\partial \lambda },\\ 2\Omega \sin\phi u=-\frac{g}{a}\frac{\partial \eta }{\partial \phi },\\ \frac{\partial \eta }{\partial t}=-\frac{H}{a\cos\phi }\frac{\partial u}{\partial \lambda }\text{.} \end{array}$$

As in Cartesian coordinates these three equations have to be satisfied by the two variable functions *u*(*t*, *λ*, *ϕ*) and *η*(*t*, *λ*, *ϕ*). To derive the (*t*, *λ*) variation of *u* and *η* one has to cross‐differentiate the first and third equations in (7) with respect to *λ* and *t*, respectively, so as to eliminate *u* between them, which yields the following wave equation:

(8) $$\frac{\partial^{2}\eta }{\partial t^{2}}=\frac{gH}{a^{2}\cos^{2}\phi }\frac{\partial^{2}\eta }{\partial \lambda^{2}}\text{.}$$

The phase speed, *C*, of these waves is obtained by writing $\eta \left(t,\lambda ,\phi \right)=\hat{\eta }\left(\phi \right)e^{ik\left(\lambda -Ct\right)}$ where $\hat{\eta }\left(\phi \right)$ is a *ϕ*‐dependent amplitude which yields the following expression for the phase speed:

(9) $$C^{2}=\frac{gH}{a^{2}\cos^{2}\phi }\text{.}$$

The right‐hand side of this expression for the phase speed is constant with respect to *t* and *λ* but not with respect to *ϕ*. To understand the origin of the *ϕ*‐dependence in (9) it is instructive to rewrite it as:

(10) $$C^{2}a^{2}\cos^{2}\phi =gH\text{,}$$

from which it is easy to realize that the phase speed in (9) is simply the spherical counterpart of the planar phase speed of gravity waves *C* ^2^ = *gH*. The modification of this simple relation brought about by the spherical geometry results from the fact that a fixed zonal length Δ*x* is related to a corresponding longitude angle Δ*λ* via the latitude‐dependent relation Δ*x* = *a*cos*ϕ* Δ*λ* so dividing both sides of this relation by Δ*t* yields *C*_plane_ = *a*cos*ϕ C*_sphere_. The key point is that *C* depends on *ϕ* on a sphere, as is evident from (9), but not on a plane.

We now show that this *C*(*ϕ*) dependence on a sphere renders this wave solution degenerate. For zonally propagating waves, where time and longitude differentiations are replaced by multiplication by i*k* and −i*kC*, respectively, the first and third equations in (7) also yield, after division by i*k*, the following relation:

(11) $$Cu=\frac{g}{a\cos\phi }\eta \Leftrightarrow u=\frac{1}{C}\frac{g}{a\cos\phi }\eta \text{.}$$

To close the problem and find explicit expressions for *u*(*t*, *λ*, *ϕ*) and *η*(*t*, *λ*, *ϕ*) we now utilize the hitherto unused second equation in (7). Substituting the relation between *η* and *u* from the second equation of (7) into (11) yields:

(12) $$u=-\frac{g}{a2\Omega \sin\phi }\frac{\partial \eta }{\partial \phi }=\frac{1}{C}\frac{g}{a\cos\phi }\eta \text{.}$$

Substituting the dispersion relation (10) then yields the following differential equation for *η*(*ϕ*):

(13) $$\frac{\partial \eta }{\partial \phi }=-\frac{2\Omega a\sin\phi }{Ca\cos\phi }\eta =-\frac{2\Omega a\sin\phi }{\pm {\left(gH\right)}^{1/2}}\eta \text{.}$$

For $\eta \left(t,\lambda ,\phi \right)=\hat{\eta }\left(\phi \right)e^{ik\left(\lambda -C\left(\phi \right)t\right)}$ the differentiation on the left‐hand side (LHS) of (13) for *C*(*ϕ*) given by (9) yields the following differential equation after division through by e^i*k*(*λ* − *Ct*)^:

(14) $$\left(\frac{\partial \hat{\eta }}{\partial \phi }-ikt\frac{\partial C}{\partial \phi }\hat{\eta }\right)=-\frac{2\Omega a\sin\phi }{\pm {\left(gH\right)}^{1/2}}\hat{\eta }\text{.}$$

The secular term in this equation, i.e. the second term on the LHS proportional to *t*, has to vanish if the solution describes a zonally propagating wave at all times. On a plane, where *C* is independent of latitude, this secular term vanishes identically. In contrast, on a sphere Eq. (9) shows that *C* is latitude‐dependent so the secular term vanishes only for *k* = 0! In this case, (14) reduces to the counterpart of (3) that determines the latitude dependence of the amplitude of Kelvin waves:

(15) $$\frac{\partial \hat{\eta }}{\partial \phi }=-\frac{2\Omega a\sin\phi }{\pm {\left(gH\right)}^{1/2}}\hat{\eta }\text{.}$$

The solution of this equation is:

(16) $$\hat{\eta }\left(\phi \right)=\eta_{0}e^{\pm \frac{2\Omega a}{\sqrt{gH}}\cos\phi }\text{.}$$

The solution with the + sign increases monotonically with cos*ϕ* so the maximal amplitude occurs on the equator while the solution with the − sign has its maximal amplitude at the poles, from where it decreases monotonically towards the equator. In both cases the exponential rate at which the amplitude decreases with cos*ϕ* is given by (*gH*)^½^/(2*Ωa*) that plays the role of the radius of deformation of the planar theory. Thus, we have shown that only for *k* = 0 can a Kelvin‐like, non‐dispersive mode with *v*(*y*) *=* 0 exist on a rotating sphere. However, a mode with *k* = 0 implies that all longitudinal derivatives in system (7) are zero, and thus all time derivatives in this system must be zero as well. Only the middle equation of system (7) remains, which specifies steady geostrophic balance. Thus, this mode does not support propagation or time‐variation and is therefore not a wave.

In summary, the classical, textbook methods for deriving Kelvin waves on a plane fail when applied to a sphere. But given the strong observational evidence for such a mode, it is reasonable to suppose that the spherical LRSWE can support a mode very similar to the Kelvin wave. We now demonstrate that this is correct: the LRSWE support a mode (specifically the gravest eastward propagating inertia–gravity wave) that resembles the Kelvin wave on a plane in that *V* is in some sense small and *C* is close to *α* ^½^.

## 4 An ad hoc eastward propagating wave on the spherical Earth

We now develop an ad hoc analytic solution of the LRSWE valid for a wide range of *α* and *k* values that in many ways resembles a Kelvin wave: it is nearly non‐dispersive and propagates at the speed of gravity waves on a non‐rotating planet. We let the ad hoc solution of (6) take the form:

(17) $$\eta \left(\phi \right)=\cos^{\gamma }\phi ;\;V\cos\phi =V_{0}\sin\phi \cos^{\gamma }\phi \text{.}$$

(Note – the normalization used here is: max{*η*} = 1). The specific functional form for the ad hoc solution is based on numerical solutions of the set (6) to be presented in section 6. Substituting this form in the first equation of (6), replacing sin^2^ *ϕ* with 1 − cos^2^ *ϕ,* and requiring that both the coefficient multiplying cos^2^ *ϕ* and the *ϕ*‐independent term vanish, yields the following relations between *V*_0_ and *γ*:

(18) $$-\alpha =V_{0}\left(1+\gamma C\right);\;C^{2}+\left(1+\gamma \right)V_{0}C+V_{0}=0\text{.}$$

Substituting the form of solution (17) into the second equation in (6) yields:

(19) $$-\gamma =-\frac{1}{C}+\frac{C^{2}k^{2}V_{0}}{\alpha C}-\frac{V_{0}}{\alpha C}\sin^{2}\phi \text{.}$$

For large *γ* the term proportional to sin^2^ *ϕ* on the right‐hand side (RHS) of this expression is order *V*_0_/(*αCγ*). To show it we note that for *γ* ≫ 1, cos*^γ^ϕ* is negligibly small at all *ϕ* ^2^ > 1/*γ*, in which case sin^2^ *ϕ* ≈ 1/*γ* (the latter condition holds even for *γ* ≥1 and not only for *γ* ≫ 1). A more careful analysis stipulates also $\sqrt{\alpha }<<1$ in addition to the above conditions. Thus, requiring the term proportional to sin^2^ *ϕ* to be much smaller than 1/*C* yields the condition *V*_0_/(*αγ*) ≪ 1 for the validity of the ad hoc solution, while requiring the term to be negligible compared to the *k* ^2^ term yields the condition *C* ^2^ *k* ^2^ *γ* ≪ 1. Neglecting the sin^2^ *ϕ* on the RHS of this expression yields:

(20) $$-\gamma =-\frac{1}{C}+\frac{C^{2}k^{2}V_{0}}{\alpha C}\text{.}$$

Substituting *C* ≈ *α* ^½^ into (20) and the first equation in (18) yields explicit expressions for *V*_0_ and *γ* and when these expressions are substituted in the second equation of (18) one obtains a polynomial in *C* which provides the correction to the zeroth‐order approximation *C* ≈ *α* ^½^ , i.e. letting *C* − *α* ^½^ ≈ *C*′ with *C*′ ≪ *α* ^½^. The resulting expressions for *V*_0_, *γ* and *C* are:

(21) $$\begin{array}{cc} & V_{0}=\frac{1-\sqrt{1+\alpha k^{2}}}{k^{2}};\;\gamma =\sqrt{\frac{1}{\alpha }+k^{2}}\;\text{and}\\ & C=\sqrt{\alpha }+\frac{\sqrt{1+\alpha k^{2}}-1}{2k^{2}}=\sqrt{\alpha }\left(1+\frac{\sqrt{\frac{1}{\alpha }+k^{2}}-\sqrt{\frac{1}{\alpha }}}{2k^{2}}\right)\text{.} \end{array}$$

**Figure 1:**
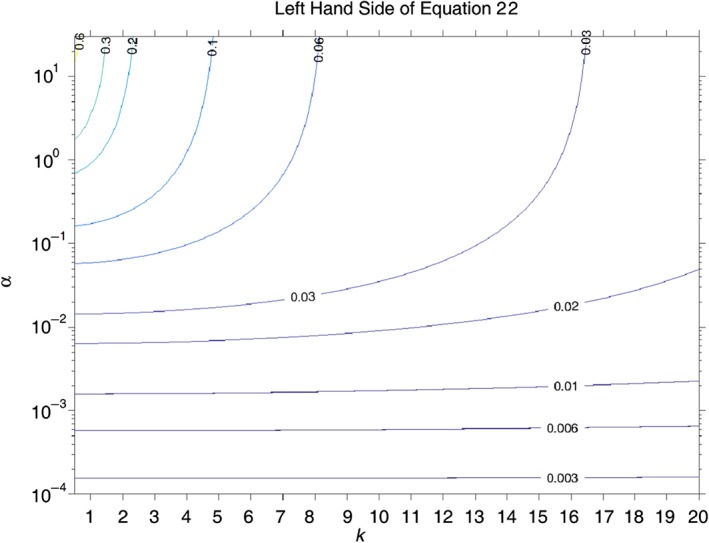
Contours of equal values of the LHS of Eq. (22) on the (α, k) plane. Clearly the inequality is satisfied even at (α = 1, k = 1). At lower values of α the LHS of Eq. (22) drops continuously. [Colour figure can be viewed at wileyonlinelibrary.com].

For consistency the expressions obtained for these parameters in this manner should satisfy either *V*_0_/(*αγ*) ≪ 1 or *C* ^2^ *k* ^2^ *γ* ≫ 1. These expressions are asymptotically correct provided (*C* − *α* ^½^)/*α* ^½^ ≪ 1, i.e. provided

(22) $$\frac{\sqrt{\frac{1}{\alpha }+k^{2}}-\sqrt{\frac{1}{\alpha }}}{2k^{2}}<<1\text{,}$$

for all *k* ≥ 1. The LHS of (22) increases monotonically with *α* and decreases monotonically with *k* (for *k* ≥ 1). Thus, the maximum value occurs at *k* = 1 and large *α* where its value is ½. Even at *α* = 1, which is far above the values relevant to Earth, the maximal value of the LHS of (22) (attained at *k* = 1) is $\left(\sqrt{2}-1\right)/2\approx 0.2$ and it drops very fast with the increase in *k*. Figure 1 clearly shows that, as expected, Eq. (22) is satisfied for all values of *k* and *α* relevant to Earth. Consistency of the various assumptions made in the derivation of this ad hoc solution also requires *γ* ≫ 1 which is satisfied when either *k* ^2^ ≫ 1 or *α* ≪ 1. In summary, the essential properties of this ad hoc solution are that: (i) it is nearly non‐dispersive, with phase speed nearly equal to the gravity wave phase speed on a non‐rotating planet; (ii) it decays quickly away from the equator as cos*^γ^ϕ*; and (iii) the meridional velocity, *v*, is small though not zero.

**Figure 2:**
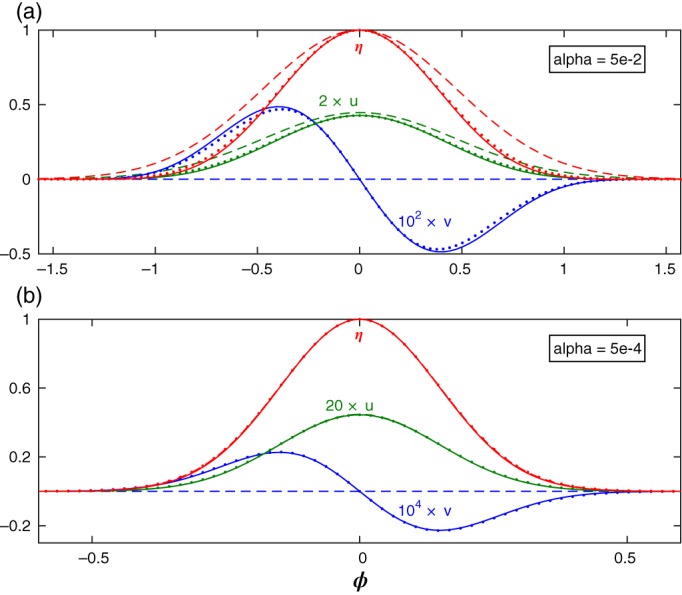
The meridional structure of u, v and η of the ad hoc mode (solid curves), the corresponding structures of the Kelvin wave in M66 (dashed curves) and exact numerical solutions of system Eq. (6) (dots) for k = 5 and (a): α = 5 × 10^−2^, (b): α = 5 × 10^−4^. For convenience the maximal amplitudes of η in both panels is taken to be 1 and the amplitudes of v and u are multiplied, respectively, by 10^2^ and 2 in (a) and by 10^4^ and 20 in (b). In both panels v is V/cos(ϕ). Note the smaller extent of the meridional domain in the abscissa in (b). The non‐dimensional time and length scale of M66 (t, y) transform to those of the present study via the relations (t, y) ⇒ ((t, y)/α ^1/4^) so the velocity transforms as u ⇒ α ^1/2^ u. [Colour figure can be viewed at wileyonlinelibrary.com].

The nature of this ad hoc mode will be examined in the following sections to determine whether it is one of the EIGn modes or a special mode that supplements the EIGn modes. However, prior to the determination of the relationship between this mode and the EIGn modes on a sphere it is instructive to compare the explicit expressions of the meridional structure of *u*, *v* and *η* with those given in M66 for *u* and *η* of the planar Kelvin waves (where *v* vanishes identically). As could be expected, the results shown in Figure 2 demonstrate that at small (baroclinic) values of *α* the ad hoc solution (solid curves) can be distinguished from M66's Kelvin wave (dashed curves) only by the presence of small, but non‐zero, meridional velocity. At large (barotropic) values of *α* the similarity between the ad hoc mode and M66's Kelvin wave is lost since the meridional structures of the former extend to high latitudes so the effect of the spherical geometry becomes pronounced. These comparisons suggest that this ad hoc solution is a modification to a sphere of M66's Kelvin wave. For comparison, (exact) numerical solutions of system (6) calculated by the method described in section 6 below are also shown (dots) in Figure 2 and they validate the accuracy of the ad hoc solution as a solution of the LRSWE on a sphere.

## 5 A Schrödinger eigenvalue equation of the LRSWE

Before turning to numerical solutions of the exact system (6) it is instructive to formulate an eigenvalue equation associated with this system whose solutions form a complete set and whose eigenvalues are closely related to the number of zero‐crossings of the eigenfunction. In contrast, solutions of system (6) are not known to form a complete set and no relationship can be established between the values of *C* and the latitudinal structure of *V*cos*ϕ* and *η*. The formulation of an eigenvalue equation associated with waves proved to be highly informative in several set‐ups including the midlatitude *β*‐plane (Paldor *et al*., 2007; Paldor and Sigalov, 2008), an equatorial channel on a sphere (De‐Leon *et al*., 2010) and the entire sphere (De‐Leon and Paldor, 2011; Paldor *et al*., 2013; Paldor, 2015). In contrast to these channel problems where the boundary conditions are ‘no normal flow’ at the channel walls, the boundary conditions that solutions of (6) must satisfy on a sphere are their regularity at the singular poles.

System (6) has two associated eigenvalue problems: the first is derived by eliminating *η* and the second by eliminating *V*cos*ϕ*. Both eigenvalue problems were derived in Paldor *et al*. (2013) but the one relevant to the issues we wish to address is obtained by eliminating *V*cos*ϕ*. Differentiating the second equation (i.e. the equation for *∂η*/*∂ϕ*) with respect to *ϕ*, eliminating both *V*cos*ϕ* and its *ϕ*‐derivative from the resulting equation by repeated application of the two equations in (6), and rearranging yields the following second‐order equation:

(23) $$\begin{array}{cc} & \alpha \frac{d^{2}\Psi }{d\phi^{2}}+\left[\underset{E}{\underbrace{k^{2}C^{2}-\frac{\alpha }{C}}}-\underset{U\left(\phi \right)}{\underbrace{\left(\sin^{2}\phi +\frac{\alpha k^{2}}{\cos^{2}\phi }\right)}}\right.\\ & \left.\;-\alpha W\left(\phi \right)\right]\Psi =0\text{,} \end{array}$$

where:

$$\begin{array}{cc} W\left(\phi \right) & =-\frac{1}{4}\tan^{2}\phi -\frac{1}{2}+\frac{3\sin^{2}\phi \cos^{2}\phi }{{\left(\omega^{2}-\sin^{2}\phi \right)}^{2}}\\ & \;+\frac{1-3\sin^{2}\phi }{\omega^{2}-\sin^{2}\phi }-\frac{2}{C}\left(\frac{\omega^{2}}{\omega^{2}-\sin^{2}\phi }\right)\text{,} \end{array}$$

and

$$\Psi \left(\phi \right)=\eta \left(\phi \right)\sqrt{\frac{\alpha C\cos\phi }{\omega^{2}-\sin^{2}\phi }}\text{.}$$

Several conclusions can be drawn directly from the formulation of the second‐order equation (23).

First, on Earth the value of *α* is about 0.05 for the entire thickness of the ocean/atmosphere and about 5 × 10^−4^ for a baroclinic atmosphere and 5 × 10^−6^ for a baroclinic ocean (where *g* is replaced by the reduced gravity) so we seek solutions of Eq. (23) in the *α* → 0 limit where *αW*(*ϕ*) is negligible compared to the O(1) potential, *U*(*ϕ*). The neglect of *αW*(*ϕ*) transforms Eq. (23) to the Schrödinger equation:

(24) $$\alpha \frac{d^{2}\Psi }{d\phi^{2}}+\left\{E-\left(\sin^{2}\phi +\frac{\alpha k^{2}}{\cos^{2}\phi }\right)\right\}\Psi =0\text{,}$$

where *E* = *k* ^2^ *C* ^2^ − *α*/*C* and in which *U* = sin^2^ *ϕ* + *αk* ^2^cos^−2^ *ϕ* is the potential. As is the case in any Schrödinger equation, (24) has infinitely many positive energy levels *E_n_*, *n* = 0, 1, 2,… and each *E_n_* is associated with three phase speeds given by the roots of the cubic $k^{2}C_{n}^{3}-E_{n}C_{n}-\alpha$. Two of these roots are the fast inertia–gravity waves whose phase speed is approximated by the roots of the quadratic $k^{2}C_{n}^{2}-E_{n}=0$ (obtained by neglecting *α*/*C*) and the third root is the slow, westward propagating, Rossby wave whose phase speed is approximated by *C_n_* = −*α*/*E_n_* (obtained by neglecting *k* ^2^ *C* ^2^).

Second, in the high‐frequency limit *ω* ^2^ > 1, the neglected potential, *αW*(*ϕ*), has no singular latitudes other than the poles, but the pole is also the singular point of the retained potential *U*(*ϕ*). Thus, all solutions of (23) with *ω* ^2^ > 1 are also solutions of (24). For *ω* ^2^ ≤ 1, solutions of (23) associated with the singularity of *αW*(*ϕ*) at sin^2^ *ϕ = ω* ^2^ can exist but these solutions are filtered out when *αW*(*ϕ*) is neglected in the transformation to (24). However, the existence of such additional low‐frequency solutions of (23) associated with the singularity of *αW*(*ϕ*) is not guaranteed.

Solutions of the Schrödinger equation (24) were derived in Paldor *et al*. (2013) for large *n* and the resulting expressions for *E_n_* from these solutions yield the following approximate expressions for the EIGn modes (see eq. 3.8 in Paldor *et al*. (2013)):

(25) $$E_{n}=\alpha {\left(n+\frac{1}{2}+\sqrt{\frac{1}{4}+k^{2}}\right)}^{2},\;n\geq 0\text{.}$$

A somewhat more accurate estimate of *E_n_* can be obtained by solving (24) numerically for *E* and *Ψ*. Once a value of *E_n_* has been found either from the approximate expression (25) or from direct numerical solutions of Eqs (23) or (24) , $C_{n}^{EIG}$ is obtained by the positive root of the cubic *k* ^2^ *C* ^3^ − *E*_*n*_ *C* − *α* (approximated by $C_{n}^{EIG}=\sqrt{E_{n}}/k$). The explicit expressions for the EIGn modes of (24) are used in the next section to classify numerical solutions of the exact system (6) with *C* > 0.

## 6 Numerical solutions of eastward propagating waves of the LRSWE

The exact system (6) can be solved numerically following the specification of boundary conditions. Specifically, the shooting method is used to calculate the dispersion relations of the eastward propagating (i.e. *C* > 0) modes that solve the exact system (6) as follows. First, any solution of (6) must satisfy the boundary condition of regularity at the singular poles, and this regularity condition can be formulated as:

(26) $$\begin{array}{c} \phi \to \frac{\pi }{2}\Rightarrow \eta ,\;V\cos\phi \to {\left(\frac{\pi }{2}-\phi \right)}^{k};\;\frac{V\cos\phi }{\eta }\to \frac{-\alpha }{kC+1},\\ \phi \to \frac{-\pi }{2}\Rightarrow \eta ,\;V\cos\phi \to {\left(\frac{\pi }{2}+\phi \right)}^{k};\;\frac{V\cos\phi }{\eta }\to \frac{\alpha }{kC+1}\text{.} \end{array}$$

(These local analyses clearly highlight the special *k* = 0 case in which *V*cos*ϕ* does not vanish at the poles so *V* is singular there. An analysis of the special *k* = 0 case is left for a future study.) Second, system (6) is integrated from *ϕ* = −π/2 + 10^−4^ to *ϕ* = 0 using the initial values of *η* = 1 and *V*cos*ϕ* given by the second line of Eq. (26). Since the functions along the diagonal of the matrix on the RHS of (6) are both odd functions of *ϕ* while the off‐diagonal functions are both even functions of *ϕ,* one of the two functions *V*cos*ϕ* and *η* must be odd while the other is even. Thus the product *η·V*cos*ϕ* is an odd function that must vanish at *ϕ* = 0 and the values of *C* are thus determined by the condition:

(27) $$F\left(C\right)\equiv {\left.\eta \cdot V\cos\phi \right|}_{\phi =0}=0\text{.}$$

**Figure 3:**
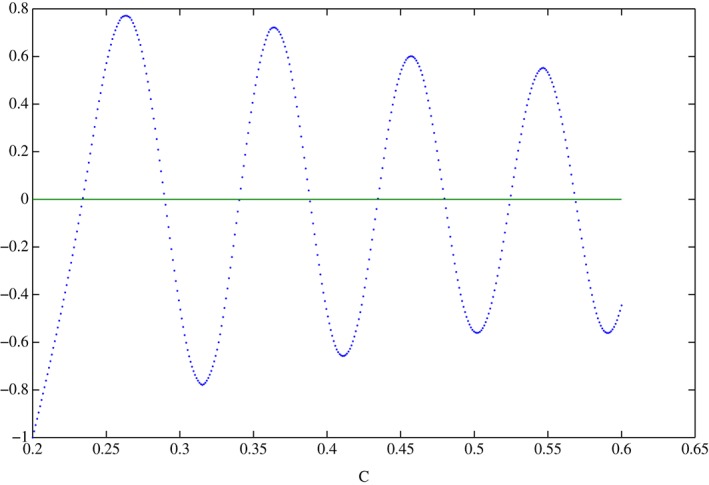
The first eight positive zeros of the function F(C) defined in Eq. (27) for α = 0.05. The results clearly show that F(C) is continuous everywhere as expected. The values on the ordinates are the product of the two functions in Eq. (27) divided by the sum of their squares to ensure that the values are O(1). [Colour figure can be viewed at wileyonlinelibrary.com].

To solve this equation we apply a zero‐finding method which was successfully used in many studies of real (i.e. one‐dimensional, where the sought frequency and phase speed are real) and complex (i.e. two‐dimensional, where the sought frequency and phase speed are complex) eigenvalue problems, e.g. Killworth *et al*. (1984), Paldor and Dvorkin (2006), Cohen *et al*. (2015). Figure 3 shows an example of *F*(*C*) calculated by integrating system (6) as described above for *k* = 5 and *α* = 0.05 (starting from a small *C* > 0) to illustrate how *F*(*C*) in (27) changes sign.

**Figure 4:**
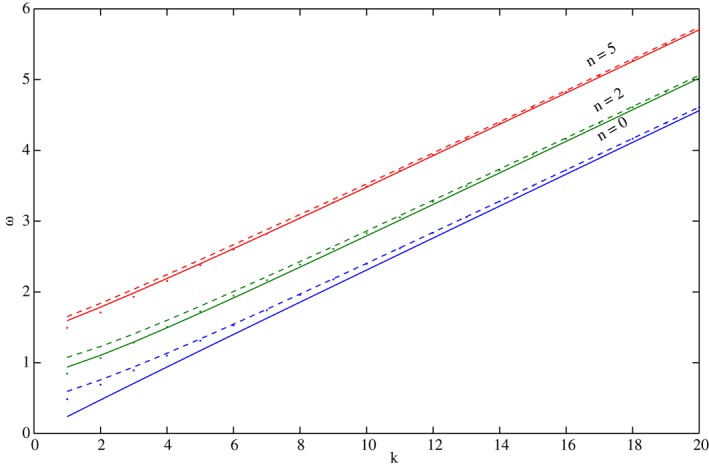
The dispersion relation ω(k) for α = 0.05 and n = 0, 2 and 5. Solid lines – numerical solutions of the exact system Eq. (6). Dashed lines – numerical solutions of the Schrödinger equation that approximates the solutions, Eq. (24), with C given by the positive root of the C(E) relation. Dots – the analytic approximate solutions for E _n_ in Eq. (25) that yield C as the positive root of the C(E) relation. Clearly, for fixed k the three estimates become close to one another as n is increased and for fixed n the same happens with the increase in k. [Colour figure can be viewed at wileyonlinelibrary.com].

When such a change‐of‐sign of *F*(*C*) occurs, a Newton–Raphson method is used to find the *C*‐value where (27) is satisfied to ten decimal digits. The convergence of this method is very fast and within 4–5 iterations, that entail changes in the fourth or fifth decimal digit of *C* only; the values of *F*(*C*) in (27) drop to the requested accuracy of 10^−9^. The values of *C* at which condition (27) is satisfied, together with the corresponding functions of *η* and *V*cos*ϕ*, are the solutions of the exact system (6). This straightforward procedure is used to determine as many solutions as needed. Figure 4 shows the dispersion relation *ω*(*k*) = *kC*(*k*) for *α* = 0.05 that corresponds to a barotropic ocean (or atmosphere). For each *k* the first (*n* = 0), third (*n* = 2) and sixth (*n* = 5) positive frequencies where (27) is satisfied are shown by the solid lines and these values are closely related to *E*‐values obtained from the Schrödinger equation (24) that yields the phase speed as the positive root of the cubic *k* ^2^ *C* ^3^ − *EC* − *α*. The values of *E* were found by numerical solution of (24) (dashed lines in Figure 4) and by analytic approximation found in Paldor *et al*. (2013) for large *n* (dots in Figure 4). It is clear from these results that all methods yield very close frequencies at large *n* and *k*. It should be emphasized that the analytic expressions (dots in Figure 4) are applied here to small values of *n* (including *n* = 0) although they formally apply only to large values of *n*. The lowest, *n* = 0, curve extends smoothly to the ω > 1 range where all waves are solutions of the eigenvalue equation (24). The calculations shown in Figure 4 for *α* = 0.05 were repeated for *α* = 5 × 10^−4^, which is more relevant to the baroclinic atmosphere on Earth and although the frequency scale in this case is an order of magnitude smaller than that of Figure 4 the results are similar – at small *k* and *n* values (that are larger here due to the two orders‐of‐magnitude decrease in the value of *α*) the mismatch is noticeable but as either *k* or *n* increases, the analytic approximation approaches the exact numerical curve.

**Figure 5:**
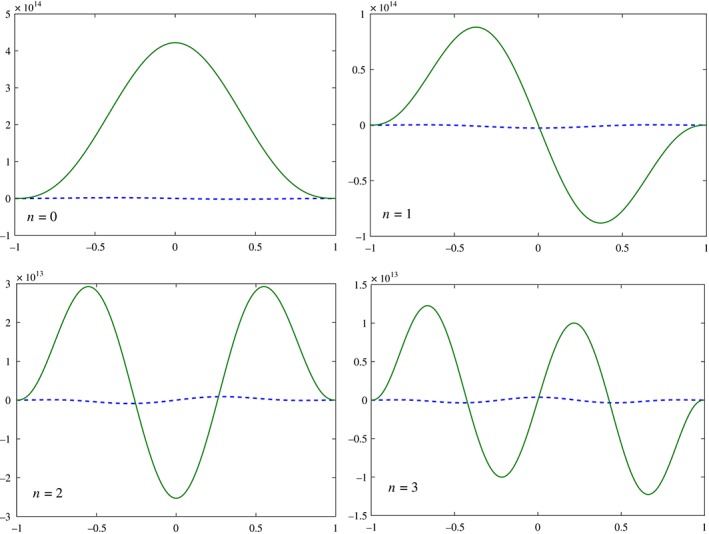
The Vcosϕ (dashed) and η (solid) eigenfunctions for k = 5 as a function of sinϕ for the values of n = 0, 1, 2 and 3 (indicated in each panel). The maximum of Vcosϕ is at least two orders of magnitude smaller than that of η in all panels. With α = 0.05 the corresponding dimensional values of Vcosϕ and η in MKS units are obtained by multiplying the non‐dimensional values of Vcosϕ by 930 and the values of η by 4400, which further increases the ratio between the maxima of η and those of Vcosϕ. [Colour figure can be viewed at wileyonlinelibrary.com].

The calculated values of *C* were validated by substituting several of them in MATLAB's ODE integrator ODE45 used for integrating system (6) from the poles to the equator and verifying that the associated *η* and *V*cos*ϕ* eigenfunctions are indeed regular at *ϕ* = ±π/2 and continuous at *ϕ* = 0. The direct integration also demonstrates the relationship between *n*, as inferred from the number of times that (27) is satisfied when *C* is increased, and the number of zeros that *η* undergoes between *ϕ* = −π/2 and *ϕ* = +π/2 (while the number of zeros of *V*cos*ϕ* is *n* + 1). The four panels in Figure 5 show the *η* and *V*cos*ϕ* functions for *n* = 0, 1, 2 and 3 and *k* = 5. It is clear from the changes of the *η*(*ϕ*) structure with *n* that this function changes as a typical solution of an eigenvalue problem even though system (6) does not appear to be an eigenvalue equation. We should point put out that although *V*cos*ϕ* is much smaller than *η* in all panels it is non‐zero at all *n*, including *n* = 0. Similar structures of the associated eigenfunctions were also calculated for *α* = 5 × 10^−4^.

The next section relates these calculations to the ad hoc, nearly non‐dispersive, mode developed in section 4 to verify whether this mode is the EIG0 or a supplementary mode to the EIGn wave type.

## 7 Comparison between the ad hoc mode(s) and EIG0 and EIG1

Having established the existence of a nearly non‐dispersive ad hoc mode in section 4 and the existence of a set of numerable EIGn modes where EIG0 is non‐dispersive in section 6, it is now possible to examine the two modes and compare them with L‐H's type‐3 and class 1 modes and with the expression derived by BZ whose phase speed matches that of the ad hoc mode in Eq. (21).

**Figure 6:**
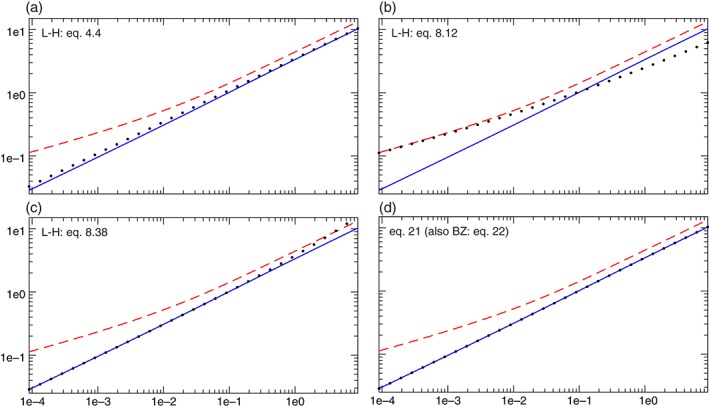
Comparison between the various expressions of the non‐dispersive mode with ω(k = 3, α) (dot markers) and the numerically computed relations for EIG0 (solid lines, blue in online version) and EIG1 (dashed lines, red in online version). (a) L‐H's class 1, n = 0 mode. (b) L‐H's type‐1, n = 0 mode. (c) L‐H's type‐3 wave. (d) The ad hoc mode found in section 4 of the present study that has the same dispersion relation as that found in equation 22 of BZ (though with entirely different meridional structure functions). Notice that L‐H's class 1, n = 0 mode in (a) and the type‐3 mode in (c) both approximate the EIG0 mode over a wide range of α. However, as is evident from their derivations, the former provides a more accurate approximation of EIG0 at large α while the latter does so at small α. [Colour figure can be viewed at wileyonlinelibrary.com].

The four panels of Figure 6 compare the dependence on α of the various analytic estimates of the non‐dispersive mode with numerical solutions of the exact system (6) calculated for *k* = 3 and *n* = 0 and *n* = 1. Panel (a) compares the two modes with L‐H's class 1 solution which is the *n* = 0 mode in the ‘slow rotation’ (i.e. large *α*) limit, and this comparison clearly shows that this mode is nothing but the EIG0 that approximates the exact solution (though with diminishing accuracy) even at *α* = 10^−4^. Panel (b) shows the comparison of the *n* = 0 and *n* = 1 frequencies of the exact system (6) with the lowest type‐1 mode of L‐H and it clearly shows that at small *α* this mode fits the *n* = 1 mode, while at large *α* this mode does not fit any of the modes of system (6). The comparison with L‐H's type‐3 mode shown in panel (c) is of particular interest since it shows that this mode is, in fact, very close to the lowest class 1 mode but its accuracy diminishes with the increase in *α* where the class 1 mode becomes more accurate. Finally, panel (d) compares the dispersion relation derived in Eq. (21) for the ad hoc mode derived in section 4, which is identical to the ad hoc BZ mode; both fit the EIG0 mode of system (6).

**Figure 7:**
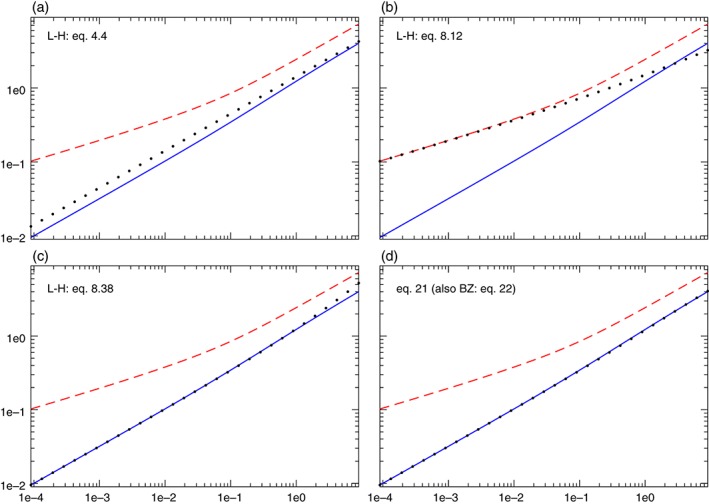
Same as Figure 6 but for k = 1. [Colour figure can be viewed at wileyonlinelibrary.com].

Figure 7 shows similar calculations to those shown in Figure 6 but for *k* = 1. Save for the less accurate fit of the class 1 wave in panel (a) and a larger range of *α* where type‐1 wave fits the EIG1 mode in panel (b), these calculations echo those of Figure 6. At larger values of *k* (e.g. *k* = 10) the difference between the frequencies of EIG1 and EIG0 becomes too small to convey a meaningful comparison between the various expressions found in recent decades for eastward propagating modes and wave solutions of the LRSWE. The conclusion that can be drawn from these results is that the ad hoc mode and the EIG0 mode are indistinguishable. The likely reason why the EIG0 mode has an ad hoc expansion while the EIGn modes with *n* ≥ 1 do not is that it is nearly non‐dispersive.

## 8 Discussion

### 8.1 Confirming that the ad hoc solution and the EIG0 are the same mode

Section 7 demonstrated that the ad hoc solution and EIG0 share the same *ω*(*α*), suggesting that the former is the EIG0 mode. As is evident from Figure 4, this mode is nearly non‐dispersive as well. As discussed in section 4 and as is evident from Figure 1, the ad hoc solution is valid as long as either *α* ≪ 1 or *k* ≫ 1, which is always the case on Earth where *α* < 0.05. We now present further evidence that the ad hoc solution and the EIG0 mode are the same mode. The (non‐dimensional) meridional velocity component is much weaker than the non‐dimensional free surface height at small alpha in both the ad hoc mode (i.e. *V*_0_ in (21) is small) and in the numerical solution (see the calculated amplitudes in Figure 5(a)). The results shown in Figure 5 demonstrate that a small ratio of meridional velocity component to free surface heights typifies all EIGn.

**Figure 8:**
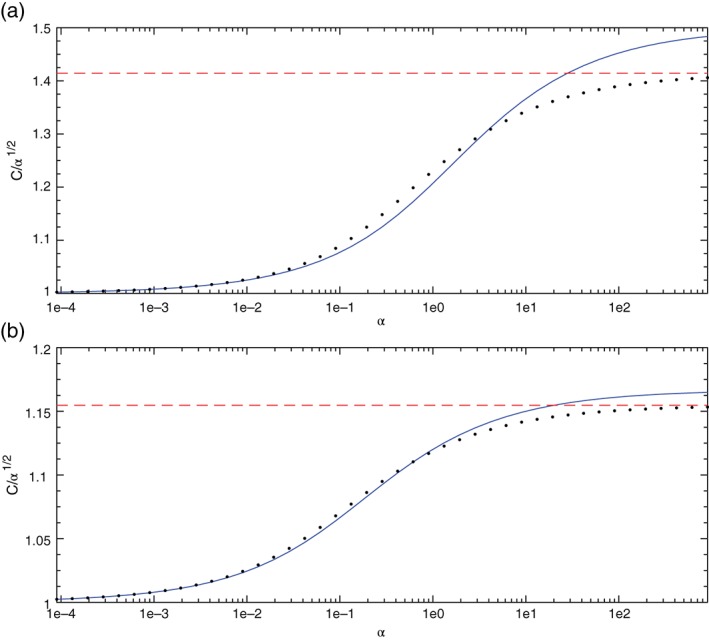
The change in the normalized phase speed C/α ^½^ of the ad hoc mode with the increase in α, i.e. when the planet's rotation frequency, Ω, is decreased. (a) k = 1, (b) k = 3. Horizontal dashed curve (red in online version): the non‐rotating limit. Solid curve (blue in online version): Eq. (21). Dots: numerical solution of n = 0 mode of the exact system Eq. (6). In both cases the non‐rotating limit is attained by the exact numerical solution, only at α of order 10^3^ but the approximate analytic expression does not converge to the non‐rotating expression even at this large α‐value. [Colour figure can be viewed at wileyonlinelibrary.com].

To further verify this conclusion we calculate in Figure 8 the changes in the phase speed of this mode (normalized on the assumed phase speed of *α* ^½^) when the planet's rotation frequency, *Ω*, is decreased, i.e. when *α* is increased. As argued in section 4, for *k* of O(1) the phase speed in (21) is valid only for small values of *α* where its normalized phase speed *C*/*α* ^½^ is close to 1. On the other hand, if this mode is indeed an inertia–gravity mode then at large *α* (∝*Ω* ^−2^) its phase speed should equal that of the non‐rotating *n* = 0 gravity mode *C* ^2^ = *α*(1 + 1/*k*), i.e. its normalized speed should approach $\sqrt{1+k^{-1}}$. Recall that the dispersion relation of the *n* = 0 gravity mode on a non‐rotating sphere is given by $\omega =\sqrt{k\left(k+1\right)\alpha }$ (*α* is the non‐dimensional counterpart of *gH*) which is approximated at large *k* (where *k*(*k* + 1) ≈ *k* ^2^) by $C=\omega /k=\sqrt{\alpha }$. Figure 8 shows the results of the calculated solution of the *n* = 0 (i.e. first zero of *F*(*C*) in (27)) along with the two asymptotic expressions – Eq. (21) and (the non‐rotating) $k\sqrt{1+k^{-1}}$ for *k* = 1 in panel (a) and *k* = 3 in panel (b). As expected, these calculations confirm that the ad hoc mode is the EIG0 mode that asymptotes the *n* = 0 gravity mode at small rotation frequency.

Additional confirmation can be obtained from the meridional structure of the ad hoc mode. Note that zonally propagating wave solutions on a non‐rotating sphere with meridional mode number *n* = 0 and zonal wave number *k* (i.e. total mode number *l* ≡ *n* + *k* = *k*) are described by the associated Legendre Polynomial $P_{k}^{k}\left(\sin\phi \right)$ which is proportional to cos*^k^ϕ* – the expression assumed in (17) for *η*(*ϕ*) in the limit of large *k* where *γ* ≈ *k* according to (21). In gravity waves on a non‐rotating sphere the velocity, *V*, is proportional to grad(*η*) so for *η* = cos*^k^ϕ* one should expect *V*cos*ϕ* to be proportional to sin*ϕ* cos*^k^ϕ* as was found above. Thus, both the meridional structure of *η*(*ϕ*) and the phase speed of the *n* = 0 non‐rotating gravity mode are identical to those of the ad hoc mode found here, which completes the verification that this ad hoc mode is a rotationally modified *n* = 0 mode of gravity waves on a non‐rotating sphere. We should also note that both gravity waves on a non‐rotating sphere and the various expressions of the ad hoc solution found here have a westward propagating counterpart with *C* = −*α* ^½^ but this mode corresponds to negative *γ*, i.e. a solution that is singular at the poles.

In summary, there is no difference between the EIG0 mode on a sphere and the ad hoc mode.

### 8.2 Comparison of the ad hoc mode to modes in BZ, M66 and L‐H

The ad hoc mode found here, from the exact set (6), generalizes the ad hoc mode derived in BZ and the type‐3 mode of L‐H to arbitrary values of *k* and for all *α* ≤ 1 without limiting the eigenfunctions to be ‘equatorially‐trapped’, i.e. without assuming sin*ϕ* ≈ *ϕ*. In contrast to the sin*ϕ* and cos*ϕ* latitudinal variation of *V*cos*ϕ* and *η* of the ad hoc mode found here, the mode found in BZ and the type‐3 wave found in L‐H both vary as a Gaussian in *ϕ*. Despite the vastly different latitudinal structure assumed here for *V*cos*ϕ* and *η,* the expression for *C* is identical to that found in equation 22 of BZ.

The identical dispersion relations obtained in this study for the ad hoc mode and that derived in BZ suggests that the eigenvalues of an eigenvalue (e.g. Schrödinger) equation are much more robust than the eigenfunctions, which is probably why the Gaussian form obtained in BZ and the trigonometric form assumed in (17) are associated with the exact same dispersion relations (see also Figure 6(d)). This identical expression provides an excellent approximation to the numerically calculated dispersion relation over a wide range of *α*‐values. The accuracy of the expressions developed in L‐H for the EIG modes are examined in Figures 6(a)–(c); panel (a) demonstrates that *n* = 0 class 1 which was developed in the slowly rotating, i.e. large *α*, limit provides an accurate approximation of the dispersion relations even for *α* ≈ 10^−3^ where the effect of rotation is not *a priori* small (though the accuracy decreases with further decrease in *α*). Similarly, panel (c) shows that L‐H's type‐3 wave, which was developed in the fast rotation, i.e. small *α*, limit provides an accurate approximation of the dispersion relation even for *α* ≈ 1 (but the error increases when *α* is further increased). When these expressions are compared with solutions of the exact system it becomes clear that both solutions approximate the EIG0 mode within their respective range of validity, but with a wide range where both of them are applicable. On the other hand, the results shown in panel (b) show that L‐H's first type‐1 mode (denoted as *ν* = 0 in L‐H's equation 8.29) provides an approximation to EIG1 only in the limit of tiny *α* but errs significantly at *α* > 10^−2^, where it does not relate to any mode. This mode approximates poorly the non‐dispersive wave shown by the solid line of panel (b).

The results shown in Figures 6(b) and 7(b) might explain what motivated L‐H to search for an additional mode only in the small *α* range (this is the type‐3, Kelvin wave) and not in the large *α* range (i.e. no class 3 wave exists). It is evident from Figures 6(b) and 7(b) that at small *α* the first type‐1 mode approximates the *n* = 1 mode and not the *n* = 0 mode so L‐H was, in some sense, ‘short’ of one mode in the small *α* limit. This may have motivated the ‘change of signature’ in eastward propagating waves noted by L‐H at the end of chapter 8 which is not encountered in westward propagating modes or in the modes of the large *α* limit.

While the meridional structure for *η* and *V*cos*ϕ* given by Eq. (17) differs from that in BZ, the sin*ϕ* relationship between *η* and *V*cos*ϕ* is common to both theories.

One of the distinguishing features of Kelvin waves on the plane (e.g. the M66 theory) is that all of the divergence arises due to the zonal wind because the meridional wind is identically zero. In contrast, as is evident from Eqs (21) and (6), for the lowest inertia–gravity mode in the spherical theory, the ratio between the contributions to the total divergence by the meridional and zonal wind components is (neglecting factors that are proportional to powers of cos*ϕ* and sin*ϕ*): $\frac{\frac{\partial V}{\partial \phi }}{ku}\approx \frac{V_{0}}{k\sqrt{\alpha }}=\frac{1-\sqrt{1+\alpha k^{2}}}{k^{3}\sqrt{\alpha }}$. By examining the RHS of this expression it can be easily verified that for *α* close to 1 and *k* near 1, the contribution of *V* approaches half of that of *u*. For large *k* or small *α* the contribution of *u* dominates the divergence. For *α* of 0.05, the ratio is 0.12 and for *α* = 5 × 10^−4^ (which is relevant to observed ‘Kelvin’ waves in the atmosphere) the ratio is 0.011 and therefore the zonal wind dominates the divergence but the contribution by meridional wind is not uniformly negligible.

In summary, while the EIG0 mode on a sphere bears several Kelvin‐like properties that justify connoting it a ‘Kelvin’ wave, it is important to distinguish between it and Kelvin waves on a plane where it *supplements* the EIGn modes.

In closing the Discussion, we refer the reader to the Appendix where we derive the Schrödinger equation of M66's as a limiting case of the counterpart equation on a sphere for *α* ^¼^ ≪ 1. However, this derivation fails when *ω* ^2^ = *k* ^2^ which implies that the Rossby waves and inertia–gravity waves found in M66 approximate the same wave types on a sphere, but M66's Kelvin waves are a fluke of the Cartesian coordinates.

## 9 Conclusions

Observational evidence for an equatorial non‐dispersive mode relevant for *k* ranging from 1 to 6 for a variety of *α* values is strong (e.g. Wheeler and Kiladis, 1999; Kiladis *et al*., 2009), and while the structure and dispersion relation of such a mode can be accurately described by M66's theory on the equatorial *β*‐plane, prior theories on the sphere were unable to find such a mode except for limited asymptotic regimes. In the present study we show that such a non‐dispersive mode does exist for all *k* and all *α* values of relevance to Earth (specifically the *n* = 0 eastward propagating inertia–gravity wave), and the phase speed of this non‐dispersive mode turns out to be nearly identical to that in the M66 theory. Hence, on a practical level, the use of the phase speed relationships derived by M66 on phase speed–wave number diagrams of tropical waves is justified, though we suggest that the label Kelvin should be demarcated with quotation marks (as in Holton and Lindzen (1968)). Furthermore, the meridional structure of the height perturbation follows *η*(*ϕ*) = cos^*γ*^ *ϕ* where $\gamma =k\sqrt{1+\frac{1}{\alpha k^{2}}}$ (see Eqs (17) and (21) and Figure 5) and therefore this mode decays rapidly with latitude so it is difficult to distinguish between it and the equatorially trapped Gaussian structure of the Kelvin wave in M66. Despite the similarity between the meridional structure and phase speed of the spherical EIG0 and the planar Kelvin wave at low *α* or large *k* it should be stressed that the former is the *n* = 0 mode of the infinite series of EIGn modes while the latter is a supplementary mode that is *not* derived from solutions of a corresponding eigenvalue equation. In addition, at large *α* and small *k*, when the meridional structure of EIG0 extends to midlatitudes, the two modes differ significantly from one another and the meridional velocity is not uniformly negligible. These findings suggest that the traditional view of Kelvin waves as a separate wave‐type that supplements the EIGn modes is valid only in planar approximations of Earth.

## Appendix 1

### Deriving the eigenvalue equation on the equatorial β‐plane as an asymptotic limit of the second order system on a sphere

A second‐order equation for *V*cos*ϕ* can be derived in a similar way to the derivation of the Schrödinger equation for *η* in section 5, i.e. by cross‐differentiation and subsequent elimination of *η* and d*η*/d*ϕ* from system (6). The resulting equation is:

(A1) $$\begin{array}{cc} & \frac{d^{2}\left(V\cos\phi \right)}{d\phi^{2}}-\tan\phi \Gamma \frac{d\left(V\cos\phi \right)}{d\phi }+\left\{\frac{\omega^{2}}{\alpha }-\frac{k}{\omega }-\frac{\sin^{2}\phi }{\alpha }\right.\\ & \;-\frac{k^{2}}{\cos^{2}\phi }-\frac{k}{\omega }\tan^{2}\phi \left(1-\Gamma \right)\}V\cos\phi =0\text{,} \end{array}$$

where $\Gamma =\frac{\alpha k^{2}+\omega^{2}\cos^{2}\phi }{\alpha k^{2}-\omega^{2}\cos^{2}\phi }$.

The transformations (*k*, *ω*) to (*α* ^− 1/4^ *k*, *α* ^1/4^ *ω*) and *ϕ* to *α* ^1/4^ *y* yield:

(A2) $$\begin{array}{c} \frac{d^{2}\left(V\cos\left(\alpha^{1/4}y\right)\right)}{dy^{2}}-\alpha^{1/4}\tan\left(\alpha^{1/4}y\right)\Gamma \frac{d\left(V\cos\left(\alpha^{1/4}y\right)\right)}{dy}\\ +\left\{\omega^{2}-\frac{k}{\omega }-\frac{\sin^{2}\left(\alpha^{1/4}y\right)}{\sqrt{\alpha }}-\frac{k^{2}}{\cos^{2}\left(\alpha^{1/4}y\right)}\right.\\ -\frac{k}{\omega }\tan^{2}\left(\alpha^{1/4}y\right)\left(1-\Gamma \right)\}V\cos\left(\alpha^{1/4}y\right)=0\text{,} \end{array}$$

where now: $\Gamma =\frac{k^{2}+\omega^{2}\cos^{2}\left(\alpha^{1/4}y\right)}{k^{2}-\omega^{2}\cos^{2}\left(\alpha^{1/4}y\right)}$.

The coefficient of the first derivative in (A2), *α* ^1/4^ tan(*α* ^1/4^ *y*)*Γ*, tends to $\alpha^{1/2}y\frac{k^{2}+\omega^{2}}{k^{2}-\omega^{2}}$ for *yα* ^¼^ ≪ 1.

For *ω* ^2^ ≠ *k* ^2^ the O(1) terms in (A2) when it is expanded in powers of *α* ^1/4^ (i.e. for *α* ^1/4^ ≪ 1) are:

$$\frac{d^{2}\tilde{V}}{dy^{2}}+\left(\omega^{2}-\frac{k}{\omega }-k^{2}-y^{2}\right)\tilde{V}=0;\;\text{where}$$

(A3) $$\;\tilde{V}\left(y\right)=V\cos\left(\alpha^{1/4}y\right)\text{.}$$

This equation is identical to equation (6) of M66 (Note that ω of the present study equals ‐ω of M66). However, for *ω* ^2^ = *k* ^2^ (i.e. when the phase speed in the original scaling is *C* ≡ *ω*/*k* = *α* ^1/2^) *Γ* is not finite so the transformation of (A2) to (A3) is not valid. Hence, in contrast to all other waves derived in M66, Kelvin waves are not an asymptotic solution of the LRSWE on a sphere and are a fluke of the Cartesian coordinates.
